# Management of rectal foreign bodies

**DOI:** 10.1186/1749-7922-8-11

**Published:** 2013-03-13

**Authors:** Ali Coskun, Nazif Erkan, Savas Yakan, Mehmet Yıldirim, Fevzi Cengiz

**Affiliations:** 1 Izmir Training and Research Hospital, Department of Surgery, Mithatpasa Cad. 964, Goztepe-Izmir, Turkey

**Keywords:** Foreign body, Rectum, Anorectal trauma

## Abstract

**Background:**

Entrapped anorectal foreign bodies are being encountered more frequently in clinical practice. Although entrapped foreign bodies are most often related to sexual behavior, they can also result from ingestion or sexual assault.

**Methods:**

Between 1999 and 2009, 15 patients with foreign bodies in the rectum were diagnosed and treated, at Izmir Training and Research Hospital, in Izmir. Information regarding the foreign body, clinical presentation, treatment strategies, and outcomes were documented. We retrospectively reviewed the medical records of these unusual patients.

**Results:**

All patients were males, and their mean age was 48 years (range, 33–68 years). The objects in the rectum of these 15 patients were an impulse body spray can (4 patients), a bottle (4 patients), a dildo (2 patient), an eggplant (1 patient), a brush (1 patient), a tea glass (1 patient), a ball point pen (1 patient) and a wishbone (1 patient, after oral ingestion). Twelve objects were removed transanally by anal dilatation under general anesthesia. Three patients required laparotomy. Routine rectosigmoidoscopic examination was performed after removal. One patient had perforation of the rectosigmoid and 4 had lacerations of the mucosa. None of the patients died.

**Conclusions:**

Foreign bodies in the rectum should be managed in a well-organized manner. The diagnosis is confirmed by plain abdominal radiographs and rectal examination. Manual extraction without anaesthesia is only possible for very low-lying objects. Patients with high- lying foreign bodies generally require general anaesthesia to achieve complete relaxation of the anal sphincters to facilitate extraction. Open surgery should be reserved only for patients with perforation, peritonitis, or impaction of the foreign body.

## Background

Rectal foreign body insertion has been sporadically described in published reports. One of the earliest case reports was published in 1919, although Haft and Benjamin referred to a case as long ago as the sixteenth century [1]. Colorectal foreign bodies (CFBs) are not an uncommon presentation to the emergency or colorectal surgery department, and some authors have suggested that the incidence is increasing [1]. Rectal foreign bodies often pose a challenging diagnostic and management dilemma that begins with the initial evaluation in the emergency department and continues through the postextraction period. Objects can be inserted in to the rectum for diagnostic or therapeutic purposes, self-treatment of anorectal disease, during criminal assault or accidents, or (most commonly) for sexual purposes [2]. Most objects are introduced through anus; however, sometimes, a foreign body is swallowed, passes thruogh the gastrointestinal tract, and is held up in the rectum [3]. Numerous objects, including billy clubs, various fruits and vegetables, nails, light bulbs, bottle, Impulse body spray cans, and turkey basters have been described as retained rectal foreign bodies. Because of the wide variety of objects and the variation in trauma caused to local tissues of the rectum and distal colon, a systematic approach to the diagnosis and management of rectal foreign bodies is essential [4]. One of the most common problems encountered in the management of rectal foreign bodies is the delay in presentation, as many patients are embarrassed and reluctant to seek medical care [4]. Most of these patients present to the emergency room after efforts to remove the object at home. Moreover, in the emergency room, patients may often be less than truthful regarding the reason for their visit, leading to extensive workups and further delays [4]. Even after extraction, delayed perforation of or significant bleeding from the rectum may occur. Hence, a stepwise approach that includes diagnosis, removal and postextraction evaluation is essential [4].

## Materials and methods

In this retrospective study, we reviewed the medical records of patients with foreign bodies in the rectum between 1999 and 2009 at Izmir Training and Research Hospital. Information regarding the foreign body, clinical presentation, laboratory and radiologic evaluation were documented. Also, patients with rectal foreign body were evaluated according to the treatment strategies, pre and postextraction endoscopic findings, surgical approach and postextraction follow-up and complications. We made a post extraction protocol that consisted of observation, repeat abdominal physical examination, a flexible rectosigmoidoscopy and repeat plain films to examine for evidence of injury and perforation that may have occurred during the extraction process. In all patients, routine abdominal x-ray examination and postextraction endoscopy were made. If there was any mucosal injury or bleeding, the patients were reevaluated by flexible rectosigmoidoscopy to rule out complete healing. This retrospective study was approved by Izmir Training and Research Hospital ethical committee.

## Results

In our study, the number of patients with rectal foreign body was fifteen.All patients were males, and their mean age was 48 years (range, 33–68 years). Information about the length of time between insertion of the foreign body and presentation at hospital is recorded in all cases. The time to presentation and removal of foreign body is a range of 6–72 h with a mean of 23, 1 h. Most of the patients were admitted to emergency room with complain of rectal bleeding, anorectal pain In one of our cases, the patient presented with hypotension, fever, tachycardia, tachypnea and abdomino-pelvic pain that lead the suspect of acute abdomen due to perforation. Physical examination revealed rebound tenderness, muscle rigidity in lower abdomen In other patients, abdominal physical examination was within normal limits. Laboratory evaluation showed elevated white blood cell count in 8 of 15 (% 51) patients. We only used abdominal X-ray to show the rectal foreign body and free air for perforation since this radiological tool was enough to rule out the diagnosis. We did not need any additional radiological investigations as CT. In our study, 12 of 15 patients examinations showed a rectal foreign body that could be reached by digital examinations. Since that, we did not use flexible rectosigmoidoscopy in these patients. In low located rectal foreign bodies, it is amenable to transanal extraction using one of many clamps and instruments. In other three patients, one of them with acute abdomen due to perporation was underwent emergency surgery without any preoperative rectosigmoidoscopy. The two of three patients need a rectosigmoidoscopy to make diagnosis for highly located foreign body in proximal rectum or distal sigmoid colon.

**Figure 1:**
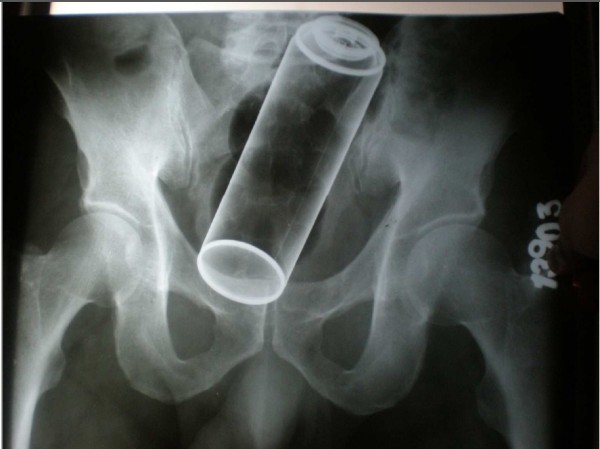
Rectal ımpulse body spray can on abdominal plain film.

The objects in the rectum of these 15 patients were an impulse body spray can (4 patients), a bottle (4 patients), a dildo (2 patient), an eggplant (1 patient), a brush (1 patient), a tea glass (1 patient), a ball point pen (1 patient) and a wishbone (1 patient, after oral ingestion) (Figure 1). Twelve objects were removed transanally by anal dilatation under general anesthesia. Three patients required laparotomy. In 2 of these 3 patients the object was lying high in the rectosigmoid colon. Objects were removed transanally by abdominal manipulation. One patient had an intraperitoneal recto-sigmoidal perforation. The perforation was treated by primary suture and proximal colostomy. Routine rectosigmoidoscopic examination was performed in all patients after object removal. and 4 had lacerations of the mucosa in the rectum. The postextraction radiological evaluation by abdominal X-ray did not show any pneumoperiteneum or retained foreign body. Oral feeding was started after rectal bleeding was stopped, and patient was stabilized. The patients were discharged up on complete clinical improvement. There was no mortality.

## Discussion

Colorectal foreign bodies are not an uncommon presentation to the emergency or colorectal surgical department. Although retained rectal foreign bodies have been reported in patients of all ages, and ethnicities, more than two-thirds of patients with rectal bodies are men in their 30 s and 40 s, and patients as old as 90 years were also reported [4]. However, there is a bimodal age distribution, observed in the twenties for anal erotism or forced introduction through anus, and in the sixties mainly for prostatic massage and breaking fecal impactions [3]. Males are commonly affected [3,5]. A useful classification of rectal foreign bodies has been to categorize them as voluntary versus involuntary and sexual versus nonsexual. One of the most common category of rectal foreign bodies is objects that are inserted voluntarily and for sexual stimulation.The foreign bodies commonly reported were plastic or glass bottles, cucumbers, carrots, wooden, or rubber objects. Other objects reported are bulb, tube light, axe handle, broomstick, vibrators,dildos,a turkey buster,utensils, Christmas ornaments [3-5]. Involuntary sexual foreign bodies are almost exclusively in the domain of rape and sexual assault. One of the most common type of rectal foreign body is best known as body packing and is commonly used by drug traffickers [4]. Involuntary nonsexual foreign bodies are generally found in the elderly, children, or the mentally ill. The objects are usually retained thermometers and enema tips; aluminum foil wrapping from pill containers; and orally ingested objects, such as tooth picks, chicken bones, plastic objects such as erasers or pill bottle caps, and even coins or small plastic toys [4]. The objects can cause severe injury. Therefore, all retained rectal foreign bodies should be treated as potentially hazardous [4].

They may complain of vague abdominal pain, rectal bleeding or pain and sometimes constipation [3-5]. Signs of infection or perforation may be evident in complicated cases. Physical examination should include a careful abdominal examination to assess for signs of peritonitis or the ability to palpate an object transabdominally. The rectal foreign body can be palpated in either the left or right lower quadrant of the abdomen. Rectal examination is essential in the diagnosis, but it should be performed after X-ray abdomen to prevent accidental injury to the surgeon from sharp objects. The foreign body may be palpable in the distal rectum. Bright red blood per rectum is often seen but is not always present. Careful attention should also be paid to the status of the sphincter, especially in patients without a prior history of foreign body placement and in those nonvoluntary cases In patients without sphincter injury, the rectal sphincter may have increased tone secondary to muscular spasm as a result of the foreign object. The sphincter may have obvious damage with visible injury to both the internal and external sphincter and should be carefully examination [4].

**Figure 2:**
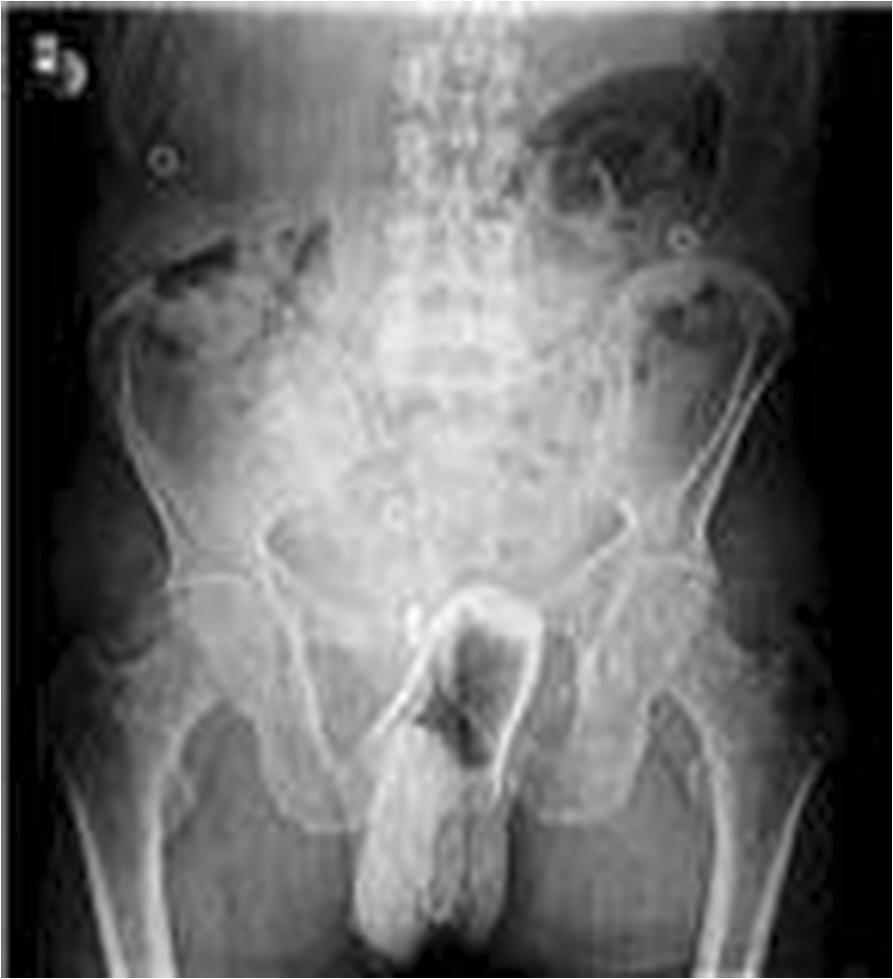
Rectal tea glass on abdominal plain film.

Laboratory evaluation is not very helpful in the patient with a rectal foreign body. If the patient has a suspected perforation, the white blood cell count may be elevated and acidosis may be present on chemistry. These laboratory tests are not very helpful, as the physical examination will be more revealing as to the extent of injury. Laboratory tests should be limited to those that are necessary in case an operation is needed. Radiologic evaluation is far more important than any laboratory test. Routine antero-posterior and lateral x- rays of the abdomen and pelvis should be obtained to further delineate the foreign body position and determine shape, size, and presence of pneumpperitoneum (Figures 1 and 2).

The first step in the evaluation and management of a patient with a rectal foreign body is to determine whether or not a perforation occurred. When a perforation is suspected, it should be determined as soon as possible whether the patient is stable or unstable. Hypotension, tachycardia, severe abdominopelvic pain, and fevers are indicative of a perforation. If there is freeair or obvious peritonitis indicating a perforation, then the patient needs immediate resuscitation with intravenous fluids and broad-spectrum antibiotics. A Foley catheter and nasogastric tube should be placed, and appropriate blood samples should be sent to the laboratory. If the patient appears stable and has normal vital signs and a perforation is suspected, a computed tomographic (CT) scan often helps determine if there has been a rectal perforation. When a foreign body is removed or absent in the rectal vault, rigid proctoscopy or endoscopic evaluation may reveal the rectal injury or the foreign body located higher in the rectosigmoid [4].

In clinically stable patients without evidence of perforation or peritonitis, the rectal foreign body should be removed either in the emergency department or in the operating room, if general anesthesia is needed. Depending on the size and shape of the object various methods have been described. Most objects can be removed transanally, and if not, then a transabdominal approach is used [3,4,6]. The authors recommend direct visualization with rigid proctoscopy or flexible sigmoidoscopy for all patients after the object has been removed to evaluate the status of the rectum and rule out ischemia or wall perforation [4].

When attempting to remove a rectal foreign body transanally, the most important factor in successful extraction is patient relaxation. This can be achieved with a perianal nerve block, a spinal anesthetic, or either of these in combination with intravenous conscious sedation [4,5]. After the patient has been appropriately sedated and anesthetized should attempts be made to remove the object. The high lithotomy position in candy cane stirrups facilitates removal of most objects and has the added benefit of allowing for downward abdominal pressure to aid in extraction of the foreign body. The anal canal should then be gently dilated to 3 fingers’ breadth. If the foreign body can be easily palpated, it is amenable to transanal extraction using one of many clamps and instruments. After successful removal of a rectal foreign body, the mucosa of the colon and rectum needs to be examined. A rigid sigmoidoscopy is recommended, although some advocate a flexible sigmoidoscopy. A repeat plain film of the abdomen is often warranted to ensure that no perforation took place during the extraction process [3-7].

Many ingenious methods have been described in literature to extract rectal foreign bodies, including Foley catheter, Sengstaken-Blakemore tube, obstetrical forceps and vacuum extractor [5]. The best method for the removal of a blunt object is to grasp to object using one of the clamps mentioned earlier or better yet, using the surgeon’s hand depending on the laxity on the canal and the success of the anal block. If the patient has a lax anal sphincter, there is a good block and the patient is adequately sedated then the object is often easily. Some smooth foreign bodies create a seal with the rectal mucosa. In this case ıt has been shown that placing a Foley cathater alongside the balloon above it helps in extraction [4,6,8-10]. Obstetric vacuum extractors have been described to grasp the object widen the anal canal and release the rectal seal [4]. Removal of the sharp objects can prove even more difficult, as they pose an additional risk for both the patient and the surgeon. These objects should be removal with the most care under direct visualization through a rigid or flexible endoscope. Once again, the rectal mucosa must be closely examined for tears, bleeding and perforation [4].

The ingestion of illicit drugs in small packets poses a particularly challenging dilemma as the surgeon has to balance extracting the foreign object with using too much force that could result in the rupture of the packets. Clamps are not recommended when attempting to remove these, as the packets are easily ruptured. Should signs or symptoms of perforation or drug ingestion/toxicity be observed, then exploratory laparotomy for removal of the remaining packets and aggressive medical treatment for the overdose is warranted.

Flexible endoscopy is reserved for objects that are located more proximally in the rectum or the distal sigmoid colon. Endoscopy also provides excellent visualization of the mucosa to evaluate for subtle and gross changes in the rectal mucosa. Endoscopy can serve as a middle ground in many cases to avoid surgical exploration by enabling evaluation and therapeutic removal of objects that may have been nonamenable to transanal extraction. Once successful extraction has been accomplished, the endoscope should be passed again to evaluate the bowel mucosa for any inadvertent injuries.

If the local perianal block and sedation are unsuccessful in the emergency department, the patient needs to be brought to the operating room for a general or spinal anesthetic to aid in the removal of the object. After anesthesia has been applied and the patient is adequately relaxed, if the foreign body cannot be removed from below then a laparotomy is indicated [3,4]. Surgery is also indicated in all patients who present with perforation (free air), sepsis, or peritonitis. Some surgeons have also described laparoscopy as an aid to push the object more distally into the rectum for a transanal removal. The first step is to attempt to milk the object distally into the rectum. If this fails, then a colotomy and removal of the foreign object is needed. This colotomy can be primarily repaired. Diversion is reserved for patients with frank peritonitis and instability, perforation with extensive fecal contamination [3,4].

The most dangerous complication of a rectal foreign body is perforation. When patients present with a rectal perforation, they should at first be stabilized like any trauma patient. After stabilization, management depends on 3 factors: first, whether the patient is clinically stable or unstable, second, whether the perforation is in an intraperitoneal or extraperitoneal location, and last, whether there is significant fecal soilage or not. A good rule of thumb is to manage a rectal perforation from a foreign body are diversion, debridement, distal washout, and drainage. Unstable patients, those with multiple comorbidities, and those with significant tissue damage and de-layed presentation more often require a diversion. On the other hand, patients who present early after the insult, those with minimal tissue damage, and those with little to no contamination can be managed with primary repair and washout. Small extraperitoneal injuries can also be managed with observation, avoidance of oral feeding, and antibiotics. However laparoscopic approach has been successfully aplied in the treatment of colonic perforations, where equivalent operative outcomes as open procedures can be accomplished in selected patients [11].

Postremoval observation depends on several factors, such as the clinical status of the patient, comorbidities, delay in presentation, and whether or not there was any resultant trauma to the rectum or surrounding tissue. Postextraction endoscopy and plain radiographs are a must before discharging any patient who had a foreign body removal [3-5]. Even with routine transanal extraction, the authors recommend several hours of close observation with serial abdominal examinations and plain films as indicated. Bleeding from lacerations in the rectal mucosa are generally self-limited. Death from sepsis and multisystem organ failure has been reported. Traumatic disruption of the anal sphincter can result in mild to severe fecal incontinence, depending on the degree of the injury. Attempts for surgical correction of any sphincter injury should be delayed until adequate time has passed to evaluate any resultant defect and clinical symptoms.

## Conclusions

Rectal foreign bodies present a difficult diagnostic and management dilemma. This is often because of the delayed presentation, wide variety of objects that cause the damage, and the wide spectrum of injury patterns that range from minimal extraperitoneal mucosal injury to free intraperitoneal perforation, sepsis, and even death. The evaluation of the patient with a rectal foreign body needs to progress in an orderly fashion, with appropriate examination, laboratory and radiographic evaluation, and resuscitation with intravenous fluids and antibiotics. In the nonperforated stable patient, the object should be removed in the emergency department with a local block and/or conscious sedation via the transanal approach. If this fails, then the patient should go to the operating room for a deeper anesthetic and attempt at transanal extraction. Surgery with a laparotomy should be reserved for patients with perforation or ischemic bowel or cases of failed transanal attempts. After removal of the foreign body, the authors suggest a period of observation, a rigid or flexible endoscopy to evaluate for rectal injury, and repeat plain films to examine for evidence of injury and perforation that may have occurred during the extraction process. Patient was referred to the psychiatrist for his perversion disorder, which was also mandatory for preventing reurrences.

## Consent

Written informed consent was obtained from the patient for publication of this report and any accompanying images.

## Competing interest

The authors declare that they have no competing interests.

## Authors’ contributions

AC, NE, SY, conceived of the study and participated in its design and coordination. MY, FC made substantial contributions to data acquisation and conception of manuscript and drafted and designed the manuscript. All authors read and approved the final manuscript.
